# The effectiveness of the Uchida-Kraepelin test for psychological stress: an analysis of plasma and salivary stress substances

**DOI:** 10.1186/1751-0759-3-5

**Published:** 2009-04-03

**Authors:** Koreaki Sugimoto, Aya Kanai, Noriaki Shoji

**Affiliations:** 1 Division of Psychosomatic Medicine and Oral Medicine, Tohoku Fukushi University, Sendai, Japan; 2 Division of Oral Diagnosis, Tohoku University Graduate School of Dentistry, Sendai, Japan

## Abstract

**Background:**

The hypothalamic-pituitary-adrenocortical (HPA) axis and sympathetic adrenomedullary (SAM) system are the major stress-response pathways. Plasma adrenocorticotropic hormone (ACTH) represents HPA axis activity, while plasma catecholamines are used as markers of the SAM system. Salivary alpha amylase (AA), chromogranin A (CgA), and immunoglobulin A (IgA) are candidate markers of stress activation, although their role has not been established. The Uchida-Kraepelin (U-K) test is a questionnaire that requires intense concentration and effort, and has been used as a tool to induce mental stress. However, it is not clear whether or not the test is effective as a psychological/mental stressor.

**Methods:**

In this study, normal young women took the U-K test and serial measurements of plasma ACTH and catecholamines (dopamine, noradrenaline, and adrenaline) (n = 10), as well as salivary AA, CgA, and IgA (n = 16) before, during and after the test.

**Results:**

We found no changes in any of these parameters at any time point during or after the U-K test.

**Conclusion:**

Our findings indicate that the U-K test is not a suitable for measuring the psychological/mental stress of young women because the plasma data showed that it did not affect the HPA axis and SAM system. The U-K test should be employed carefully as a psychological/mental stressor due to insufficient scientific evidence of its effectiveness. In addition, salivary AA, CgA, and IgA should not simply be compared with previous reports, because the mechanism of secretion and normal range of each salivary parameter remain unknown. Salivary AA, CgA, and IgA may not be suitable candidate markers of psychological/mental stress.

## Background

The Uchida-Kraepelin (U-K) test is a questionnaire modified from the Kraepelin's arithmetic test, which was originally developed by Y. Uchida [1]. The U-K test measures the ability of takers on task performance speed and task performance accuracy. The results of the U-K test provide an estimate of the individual's character. The U-K test requires focused effort and attention by the subject, making this test useful for the assessment not only of character but also of mental stress [2,3]. In fact, it has been used widely to assess work aptitude as a mental stressor [2,4-12]. However, it is not clear whether or not the U-K test *per se *provides psychological/mental stress to its taker.

Homovanillic acid (HVA) is a major metabolite of dopamine [11,12], and 3-methoxy-4-hydroxyphenylglucol (MHPG) is a metabolite of noradrenaline [3]; both have been used as markers of stress incurred by the U-K test. However, serum catecholamines; dopamine (DA), noradrenaline (NA) and adrenaline (AD) have not been measured as stress markers, although excitement up-regulates these molecules, especially NA and AD, through the sympathetic nervous system [7,13-16]. Likewise, the HPA axis marker adrenocorticotropic hormone (ACTH) has not been used as a marker of psychological/mental stress in a U-K test study, although the hypothalamic-pituitary-adrenocortical (HPA) axis is also known to be stimulated by various stressors [7,15,16].

Salivary fluid is often sampled for biological assays because of ease and noninvasive accessibility. Several salivary markers have been proposed as biological markers of psychological stress in humans, including salivary α-amylase (sAA) [2,7,17-24], salivary chromogranin A (sCgA) [25-29], and salivary immunoglobulin A (sIgA) [27,30-36]. However, none are yet established as routine stress markers.

In the present study, we measured plasma ACTH, DA, NA and AD to investigate the effects of the U-K test applied as a psychological/mental stressor on the HPA axis and sympathetic adrenomedullary (SAM) system. We also examined the effects of the U-K test on sAA, sCgA, and sIgA secretion.

## Methods

### Participants and experimental design

**Figure 1:**
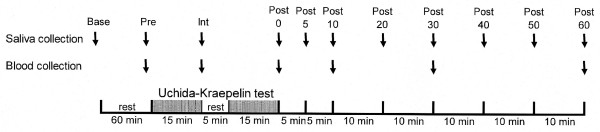
**Experimental design**. Saliva and blood samples were collected at 11 and 6 different time points, respectively, both each 5 min. Arrows indicate the start point of collecting.

A total of 19 healthy young women (age, 19 to 23 years) participated in the present study. None were on medication, had bouts of gingivitis, drank alcohol more than twice per week, smoked or was pregnant. Figure 1 shows the study design. On the day of the study, the volunteers had lunch then rinsed their mouth and rested for 60 min, after which they abstained from food and drink. Saliva was collected from 16 volunteers and blood was collected from 10 volunteers before, during and after the U-K test presented on a compact disc player (Japan Psychiatric Technology Institute Inc.) in the afternoon between 2 and 3 PM. All saliva and blood samples were taken at the same time of the day for each volunteer.

The U-K test is a serial addition test, which requires takers to perform calculations as fast and accurately as possible within 30 min. This was achieved using pre-printed paper containing 15 lines of random, single-digit, horizontally aligned numbers. For each minute of the test, the subject was instructed to begin a new line regardless of their position on the current line. Each line contained an excess of calculations such that the subjects were not able to finish any line for a particular minute before being prompted to move on to the start of the next minute by the examiner's prompting. This test is usually performed for repeated 15 min of work and 5 min rest cycles.

To investigate the participants' character, the MOS 36-Item Short-Form Health Survey (SF-36) [37] was given to all participants before the U-K test. SF-36 version 2 includes scales for physical functioning, physical role, bodily pain, social functioning, general health perceptions, vitality, emotional role, and mental health. This SF-36 can be used to check health-related quality of life. Furthermore, the data of the U-K test of each participant was correlated with each character and with plasma and salivary stress substances. The character results obtained by the U-K test are composed of behavioral activation, mood or behavioral variation, behavioral sensitivity, character or behavioral deviation/tendency, and total typical/atypical manner.

The Ethics Committee of Tohoku Fukushi University approved the study protocol. The study was carried out in compliance with the Helsinki Declaration. All participants were fully briefed on the scope of the experiment, and informed consent with oral explanation and written documents was obtained from each participant prior to the study.

### Measurements of plasma ACTH, dopamine, noradrenaline, and adrenaline levels

A cannula was inserted in a vein of the left arm and maintained via a heparin-saline lock system 3 h before undergoing the U-K test. Four ml of blood was collected over 5 min via the cannula immediately before, during, and at 0, 10, 30, and 60 min after the test. Plasma was prepared and assayed for ACTH by radioimmunoassay (RIA kit RK-001-01, Phoenix Pharmaceuticals Inc, Burlingame, CA), while DA, NA, and AD concentrations were estimated by high-performance liquid chromatography (HPLC).

### Saliva collection and salivary flow rate calculation

Saliva was collected by the spitting method, which involves collecting spontaneous/passive drooling saliva, at baseline (at the start of the rest period), immediately before, during, and at 0, 5, 10, 20, 30, 40, 50, and 60 min after the test. Saliva was collected over 5 min and kept on ice. Samples were centrifuged at 1,500 × *g *for 15 min and the supernatant used for CgA and IgA measurements. The remainder was stored frozen at -80°C until further use. The amount of collected saliva in grams was converted to milliliters assuming that the specific gravity of saliva is 1.01 (g/ml); this value was divided by 5 to ascertain the salivary flow rate in ml/min.

### Salivary α-amylase, chromogranin A, and immunoglobulin A assays

Salivary AA was measured using a Cocoro meter^® ^(Nipro Co, Osaka, Japan) [7,38] within 2 min after sampling. The meter tip was immersed in saliva under the tongue of the subject for 30 sec starting from 1.5 through 2 min after each time point of saliva collection. This amylase monitor uses a dry chemistry system, measuring enzymatic activity in a batch type, and using a reagent paper containing the amylase substrate, 2-chloro-4-nitrophenyl -4-*O*-β-D-galactopyranosylmaltoside (Gal-G2-CNP, Toyobo Co, Tokyo).



Amylase hydrolyzes the Gal-G2-CNP present in the reagent paper to produce a color change from white to yellow, measured optically for 30 sec. One unit of α-amylase activity (U) was defined as the enzymatic activity that could produce reduced sugar equivalent to 1 μmol maltose at 37°C in 1 min. Salivary AA was expressed in U/ml [38].

Salivary CgA and IgA were measured by enzyme immunoassay (EIA) using the YKO human chromogranin A EIA kit (Yanaihara Institute Inc^®^) and salivary secretory IgA indirect enzyme immunoassay kit (Salimetrics LLC Inc, State College, Pennsylvania, USA). This IgA kit measured salivary IgA within the range of 379.39 ± 261.47 μg/ml, (mean ± SD, n = 21).

### Statistical analysis

Data are expressed as mean ± SD. All data were analyzed by one-way analysis of variance (ANOVA) followed by *Tukey's test*. Sample numbers are 10 for blood samples and 16 for salivary samples. A *P *value less than 0.05 denoted the presence of a statistically significant difference.

## Results

### The U-K test does not influence plasma ACTH concentration

**Figure 2:**
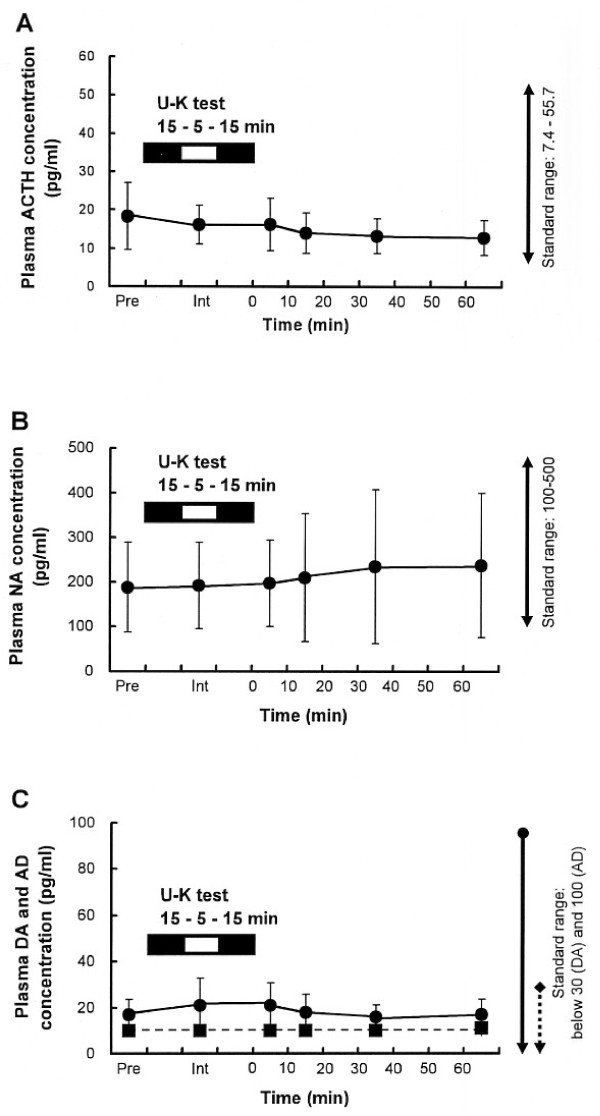
**Plasma concentrations of [A] ACTH, [B] noradrenaline, [C] dopamine and adrenaline before and after the U-K test**. Plasma ACTH, NA, AD (solid line), and DA (dotted line) concentrations (pg/ml) before, during, and after the U-K test are shown as mean ± SD (n = 10). *Pre*: immediately before the test; *int*: during the test; 0, 10, 30, 60 min: minutes after completion of the U-K test. Standard ranges of plasma ACTH, DA, AD, and NA represent the reference ranges (ACTH: 7.4–55.7; NA: 100–500; DA < 30, AD < 100, pg/ml, respectively), not the normal range. U-K: Uchida-Kraepelin, NA: noradrenaline, DA: dopamine, AD: adrenaline.

To examine the effect of the U-K test on the HPA axis, plasma ACTH was measured. Plasma ACTH seems to be more sensitive than plasma cortisol to stress on the HPA axis [39]. Plasma ACTH concentrations before, during, and after the U-K test are shown in Figure 2[A] (n = 10). There was no significant change from the standard range in mean ACTH values during or after the U-K test [*df *= 5, *F*-value = 1.27, *P *= 0.29].

### The U-K test does not influence plasma noradrenaline, dopamine, and adrenaline concentrations

To examine the effect of the U-K test on the SAM system, plasma levels of NA, DA, and AD. Plasma NA, DA, and AD concentrations measured before, during, and after the U-K test are shown in Figure [2B and 2C]. There were no significant changes from the standard range of each catecholamine in NA, DA, and AD values during or after U-K test. Plasma NA: [*df *= 5, *F*-value = 0.27, *P *= 0.93]. Plasma DA: [*df *= 5, *F*-value = 1, *P *= 0.43]. Plasma AD: [*df *= 5, *F*-value = 0.66, *P *= 0.65].

### The U-K test does not influence salivary flow rate

**Figure 3:**
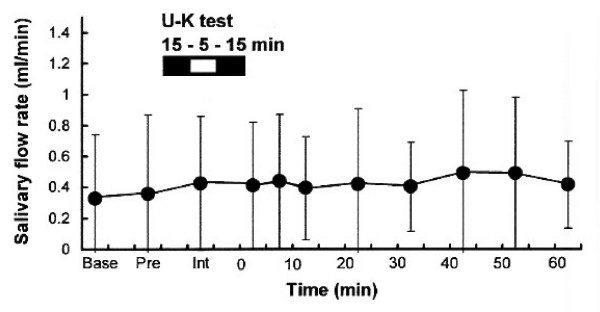
**Salivary flow rate before and after the U-K test**. Data (ml/min) are mean ± SD (n = 16). Saliva was collected for 5 min by the spitting method. *Base*; basal state of volunteers 1 h before the U-K test; *pre: *immediately before the test; *int: *during the test interval; *0, 5, 10, 20, 30, 40, 50, 60 *min: minutes after completion of the U-K test. U-K: Uchida-Kraepelin.

To rule out a possible effect of the volume of secreted saliva, salivary flow rate (ml/min) was measured. The flow rate of salivary secretion is known to influence sAA, sCgA, and sIgA concentrations [22,40]. Salivary flow rates before, during, and after the U-K test are shown in Figure 3. No significant differences in flow rate were observed in this study [*df *= 10, *F*-value = 0.21, *P *= 0.99]. Although the variability in salivary flow rate was large among individuals, we could neglect the effect of salivary secretion statistically.

### The U-K test does not influence salivary α-amylase activity, chromogranin A or immunoglobulin A

**Figure 4:**
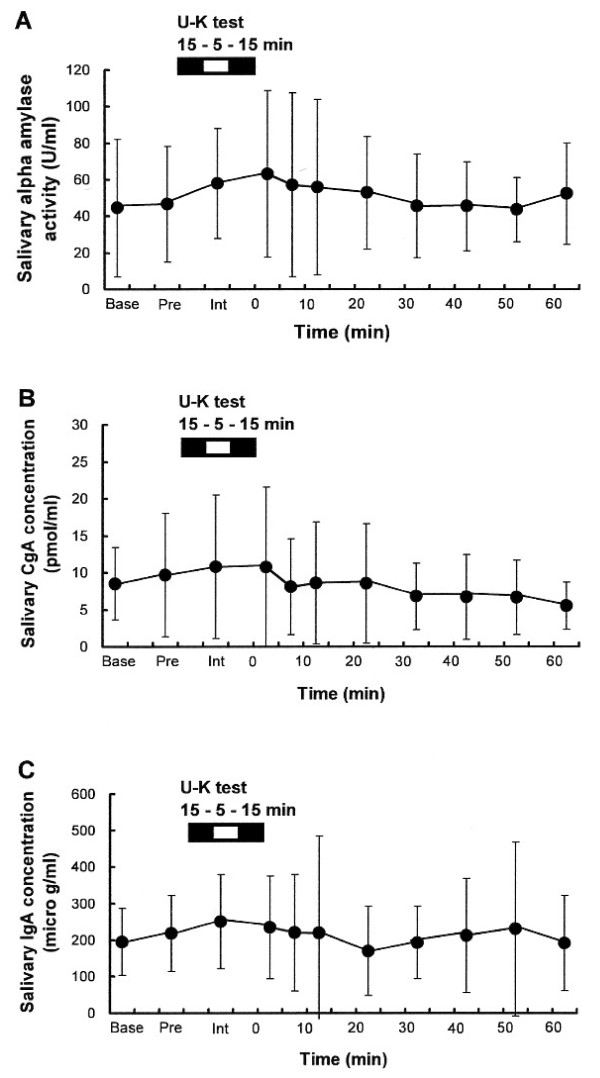
**Salivary concentrations of [A] α-amylase activity, [B] chromogranin A, and [C] immunoglobulin A before and after the U-K test**. Data ([A] U/ml, [B] pmol/ml, [C] μg/ml) are mean ± SD (n = 16). Saliva was collected for 5 min by the spitting method. *Base; *basal state of volunteers 1 h before the U-K test; *pre: *immediately before the test; *int: *during the test interval; *0, 5, 10, 20, 30, 40, 50, 60 *min: minutes after completion of the U-K test. U-K: Uchida-Kraepelin, CgA: chromogranin, IgA: immunoglobulin A.

Salivary AA activities were not significantly different between the reference range (Base) and the range before, during, and after the U-K test, as shown in Figure 4[A]. Similar findings were recorded for sCgA and sIgA concentrations before, during, and after the U-K test, as shown in Figure [4B and 4C]. Salivary AA: [*df *= 10, *F*-value = 0.56, *P *= 0.84]. Salivary CgA: [*df *= 10, *F*-value = 0.92, *P *= 0.51]. Salivary IgA: [*df *= 10, *F*-value = 0.34, *P *= 0.97]. Large inter-individual variability was noted in sAA activity, sCgA and sIgA, and there were no statistically significant changes in these parameters. These results differ from those reported previously by others; eg, the U-K test or mental arithmetic test is reported to result in increases in sAA [7] and sIgA [2,35,41].

### Psychological assessment of participants and the relationship between their character and stress substances

The psychological status of each participant was assessed by the analysis of SF-36 and the U-K test. No remarkable psychological patterns or character deviation were noted for the participants. None of the categories of the SF-36 or U-K test was correlated with plasma ACTH, DA, NA, or AD concentration or with salivary AA, CgA, or AA (data not shown).

## Discussion

The U-K test given to the young women studied induced no significant changes in any of the reported markers of psychological/mental stress, i.e., plasma ACTH and catecholamines. In general, ACTH is up-regulated through the HPA axis under stress conditions, often accompanied by secretion of AD and NA from the adrenal medulla and sympathetic nerve terminals [7,13-16]. In fact, the Trier Social Stress Test (TSST) induced up-regulation of plasma ACTH until 30–40 min after the stress [42,43], although plasma DA, NA and AD have not been measured as stress markers. Therefore, it seems that the U-K test did not affect the HPA and/or SAM system of the participants of our study. Dickerson and Kemeny [39] described that though acute psychological stressors can elicit cortisol and ACTH activation, not all acute psychological stressors provoke this system. There is a substantial degree of variability in the size of cortisol and ACTH effects, depending on the characteristics of the stressor. Our results do not contradict the study of Dickerson and Kemeny [39]. The U-K test may have borderline value as a stress task because they categorized the U-K test as a cognitive task in which cortisol has little effect. In this study, there may be problem in that our experiment was done with a limited population of young women. However, Takai et al. [16] recently investigated gender differences in the activities of the HPA and SAM systems in response to acute psychological stress. They found no gender differences in the resting salivary cortisol and amylase levels. Furthermore, there were no gender differences in the salivary amylase level after stressful video viewing. Taken together, we do not recommend the use of the U-K test, at least for young women, for inducing psychological/mental stress, despite many reports of such an application [2,4-12].

The mean salivary flow rate of our participants was 0.33 ± 0.41 ml/min by the passive spitting method. This rate is consistent with that reported in previous studies [20-22,24,33,44]. In this study, we used the spitting method for saliva collection in preference to salivettes^® ^cotton (Sarstedt, Numbrecht, Germany) to avoid mechanical stimulation of salivary secretion. One recent study compared the two methods and found no difference in mean saliva volume collected [45]. In contrast, another study showed that the relative increases in flow rate induced by psychological stress were significantly higher in saliva obtained by the spitting method than in that collected using salivettes cotton, although the saliva flow rate was not significantly different between the two methods under non-stress conditions [22]. Moreover, the cotton method caused substantial interference to many biomarker assays, including sIgA, giving falsely low readings due to the absorbent materials [45,46]. We therefore chose not to use the salivettes cotton method, and followed an earlier recommendation to use the spitting method for both unstimulated and stimulated whole saliva collection [47]. After all, the flow rates were not altered by the U-K test, arguing against any stress effect of the test on sAA, sCgA, or sIgA secretion.

No changes in salivary AA, CgA, and IgA concentrations were induced before during and after the U-K test. Goi et al. [2] studied the effect of the U-K test on salivary flow rate, sIgA, and sAA at short time points; 30 min and immediately prior to the test, as well as immediately and 30 min after the test. Only salivary flow rate 30 min after the U-K test and sIgA immediately after were increased over the 30-min-prior readings. The above study had a different methodology than ours; the authors used cotton for salivary collection with rough time points and a quantitative kinetic method to assay sAA.

Salivary AA is a potentially useful and easily collectable surrogate marker of the SAM system [48]. Rapid activation and recovery of sAA secretion is thought to characterize the response of the SAM system to stress [23]. Table 1 shows the levels of sAA under resting or pre-stress conditions reported in previous studies. The data show large variability among the studies. There was marked individual variation of sAA among the subjects tested [38], and relative diurnal variations reported for sAA [17,22] and sIgA [20]. Individual differences in sAA are related to age and pubertal development, and the level may be sensitive to specific experiences that involve physical, social, and cognitive demands [23]. This may explain the lack of normal range specified for sAA.

**Table 1:** Values and methods from previous reports for the determination of representative salivary stress markers during a non-stress period

**Salivary stress marker**	**Reference**	**Value**^¶^	**n**	**Measurement method**	**Salivary collection method**
sAA (U/ml)	[2]	39.1 – 309.1*	39	EKM	cotton
	
	[17]	15 – 365 (14 – 16 PM)	76	EKM	cotton
	
	[18]	60 – 200	12	EKM	cotton
	
	[48]	30 – 60	15	EKM	cotton
	
	[61]	20 – 230	12	EKM	cotton
	
	[62]	40 – 150	13	EKM	cotton
	
	[22]	9 – 50	26	EKM	Cotton
		0 – 27	26		passive spitting
	
	[16]	male 63.6 – 179.4	18	EKM	passive spitting
		female 81.1 – 183.1	14		
	
	[19]	7.8 – 76.6	20	EKM	passive spitting
	
	[20]	290 – 700	8	EKM	passive spitting
	
	[21]	21.2 – 149.8	67	EKM	passive spitting
	
	[24]	males 50.9 – 192.1	53	EKM	passive spitting
		females 58.2 – 190.3	30		
	
	[63]	0 – 50	13	EKM	passive spitting
	
	[7]	2.6 – 65.0	11	DCS**	test-strip
	
	[38]	9.5 – 43.4	15	DCS**	test-strip
	
	this report	6.9 – 82.2	16	DCS**	test-strip

sCgA (pmol/ml)	[54]	0.2 – 0.4	40	ELISA**	cotton
	
	[64]	1.2 – 2.4	11	ELISA**	cotton
	
	this report	3.6 – 13.4	16	ELISA**	passive spitting

sIgA (μg/ml)	[2]	4.65 – 15.05	39	ELISA	cotton
	
	[32]	28.7 – 92.7	27	ELISA	cotton
	
	[35]	7.6 – 132.4	27	ELISA	cotton
	
	[56]	6.4 – 17.7 (PM sampling)	8	ELISA	cotton
	
	[65]	9 – 11	16	ELISA	cotton
	
	[66]	186.7 – 653.4	18	ELISA	cotton
	
	[45]	38.7 – 79.3	10	ELISA	Cotton
		61.7 – 107.5			passive spitting
	
	[46]	0 – 100.62	8	EIA	Cotton
		114.12 – 238.78			passive spitting
	
	[20]	91.2 – 148.3	8	ELISA	passive spitting
	
	kit instruction	117.92 – 640.86	21	EIA	unknown
	
	this report	103.2 – 286.6	16	EIA	passive spitting

^¶^Values are mean ± SD.

*The mean sAA value in the original report (Goi et al., 2007) was 17.41 ± 13.50 (± SD, × 10^4 ^U/ml), however this value was probably an incorrect calculation. In this table, we multiplied the original value by 10^-3^.

** Our measurement is similar to that recommended by the manufacturers.

sAA: salivary alpha amylase, sCgA: salivary chromogranin A, sIgA: salivary immunoglobulin A, EKM: enzyme kinetic method, DCS: dry chemistry system, EIA: enzyme immunoassay, ELISA: enzyme-linked immunosorbent assay.

CgA is a major soluble protein in adrenal chromaffin cells and adrenergic neurons. It belongs to a family of highly acidic proteins, the chromogranins, which are co-stored and co-released with AD and NA in the adrenal medulla [49], and secreted with the catecholamines into the circulation [50]. Moreover, CgA is a valuable indicator of SAM activity [51]. Human CgA is produced by the submandibular glands and secreted into saliva [52]. Salivary CgA is considered a marker of sympathetic nervous activity including the SAM system [53]. Table 1 also lists the reported sCgA levels measured at rest or before stress. Our sCgA values measured during the non-stress periods were higher than those reported in two previous studies, and this difference could be due to the use of different collection methods. Moreover, the secretory pattern of sCgA follows circadian rhythms [54]. Like sAA, a normal range for human sCgA also remains to be specified.

Salivary IgA is a convenient and commonly used indicator of immune status. This parameter reportedly indicates the functional status of the entire mucosal immune system [55]. Numerous studies have shown that sIgA is sensitive to psychological variables. Early studies explored the relationship between sIgA and long-term psychological stress or in individuals particularly prone to stress [56]. Such studies consistently revealed stress-related down-regulation of sIgA [57]. This evidence supports the argument that prolonged psychological stress can compromise certain aspects of immune function [56]. Paradoxically, acute responses to a psychological challenge include a rise in sIgA concentration [58]. This mobilization of sIgA has also been reported in response to acute laboratory psychological stress tests including mental arithmetic tests [35,41]. However, there is little information on whether or not sIgA secretion is affected by psychological stress, or how that might occur mechanistically. An association was demonstrated between psychological activation of the HPA axis and the modulation of sIgA [59,60]. However, these studies showed opposite correlations of salivary cortical and sIgA response. Moreover, the non-stress sIgA values varied between studies (Table 1) [61-66]. Our results were compatible with the expected range reported in the kit instructions, making our sIgA data more reliable.

Salivary IgA is produced by plasma cells located adjacent to the salivary gland ducts and acini [67]. The sIgA response to oral antigens may be induced via two mechanisms: stimulation of the response directly following the proliferation and differentiation of local lymphoid cells, and the migration of antigen-sensitized IgA precursor B cells from gut-associated lymphoid tissue to salivary glands [67] (and references therein). Further investigations of the association between sIgA and the stress response as well as the mechanisms involved are clearly needed.

Many scientists have used the U-K test as a psychological/mental stressor. Our results indicate, however, that the U-K test should be employed carefully as a psychological/mental stressor due to insufficient scientific evidence of its effectiveness. In addition, salivary AA, CgA, and IgA are probably not suitable candidate markers of psychological/mental stress, because the mechanism of stress-induced secretion and their normal ranges remain to be specified.

## Conclusion

The U-K test has been regarded as a psychological/mental stress inducer. We measured the serial changes in plasma ACTH, DA, NA, and AD levels, as well as salivary AA, CgA, and IgA concentrations before, during and after conducting a U-K test. None of these stress substances changed in response to the test, suggesting that it does not elicit the HPA axis and/or SAM system stress response. Therefore, the U-K test does not seem to be an appropriate psychological/mental stressor. In addition, salivary AA, CgA, and IgA should not simply be compared with previous reports, because the mechanism of secretion and normal range of each salivary parameter remain unknown. Salivary AA, CgA, and IgA may not be suitable candidate markers of psychological/mental stress. Since the present experiment was done only with young women, further studies of other populations are necessary.

## Competing interests

The authors declare that no financial support or compensation has been received from any individual or corporate entity for research or professional service and there are no personal financial holdings that could be perceived as constituting a potential conflict of interest.

## Authors' contributions

KS and NS designed the protocol, performed the statistical analysis, and wrote the manuscript. AK and KS carried out the assay of plasma and salivary stress substances. NS and others participated in the human experiment.
